# Supplementary material for: TWINKLE: An open-source two-photon microscope for teaching and research
Source: PLoS One. 2025 Feb 13;20(2):e0318924. doi: 10.1371/journal.pone.0318924 (PMC11824991; doi:10.1371/journal.pone.0318924)
Supplement: Attachment — Submitted filename: Reply to Reviewers.pdf [file pone.0318924.s002.pdf]

Dear Editor and Reviewers:

We would like to thank the reviewers for their time, and their assessment of our work being a "*valuable contribution*" that is "*well written and easy to follow*" while also "*inspiring and thought-provoking*" for more experienced researchers. In incorporating their suggestions, we believe that the manuscript has profited both in clarity and scope.

To address the reviewers' concerns, we have made changes to the text (indicated in blue), **Figure 1** and **Supplemental Figure 11**. In the text below, we provide brief comments on how these changes reflect the reviewers' comments.

Reviewer #1: The manuscript reports "Twinkle": a microscope for Two-photon Imaging in Neuroscience, and Kit for Learning and Education, which is described as a fully open, high-performance and cost-effective research and teaching microscope. The text is well written and easy to follow also for more inexperienced scientists, and the basic idea can be of interest. The supplementary guide is very detailed and I find it very useful. However, I find some criticalities.

The major one is that the overall cost for all the instrumentation is not much cheaper than many other custom-made two-photon microscopes available in optic laboratories, especially since the price does not consider the costs for laser, the most expensive component. Therefore, I do not see a novelty in this, however I find more on the ease of production and the educational nature of the paper.

We thank the reviewer for articulating their concerns.

(1) First, we would like to make it explicit that we never claimed that our microscope was "cheap". We consciously chose the words "*cost-effective*" and "*at reasonable cost*" to communicate that we are aware of the expenses associated with the various parts. We believe that we made reasonable choices with cost in mind, but the instrument is certainly a high-performance device. This necessitated certain expensive choices over cheaper alternatives (such as the large diameter of the optical components; GaAsP PMTs; etc.). To communicate this more clearly, we have added specific details to the abstract. In addition, we have adopted the reviewer's choice of words in the revised abstract and write: "*It is a fully open, high-performance and easy-to-set-up microscope that can effectively be used for both education and research.*"
(2) In the new introduction, we have stressed the ease of production, the educational nature and point to the "*very detailed*" instructions in the supplement.

My first suggestion is to introduce more details on how the design of the setup can be improved/modified based on other specific uses that one can need it for, as the main purpose of the paper is to be educational.

The reviewer raises two points that they wish to be highlighted in the article: (A) education and (B) improvements/modifications to the instrument.

To point (A), we have made the following additions to the paper:

(1) We added descriptions and references to misalignments and resulting aberrations.
(2) We added a note on microscope characterization and its importance.
(3) We added a sketch of the derivation of **Eqn. (2)** to the supplemental material (the derivation of pulse width as function of dispersion).
(4) We expand on the use of doublets over singlet lenses.

To point (B), we had already included a section on improvements and modifications, with nine specific suggestions. We have further expanded this section, and now discuss 13 changes to the design. We have added:

(5) A note on remote focussing and electrically tunable lenses, and when these alternatives to the piezoelectric collar could be used.
(6) A note on field curvature. In neuroscience, field curvature is often not as important as samples are typically curved. If the microscope was used for other purposes, for example precision measurements of *ex vivo* tissue samples, or flat samples in material science, this could be further optimized.
(7) A note on resolution. When working with fixed samples, movement artifacts are not an issue. It would then be strategic to fully fill the back aperture of the objective and produce a PSF that is as tight as possible.
(8) A note on resonant galvos. These are very fast, but cannot be steered. If a user was interested in imaging custom ROIs, different mirrors would be more useful.

The following are questions that arose while reading the manuscript and may suggest additional information or clarifications that could strengthen the paper.

1. Please mention, **in the introduction section**, how this setup can be applied not only in neurosciences, but also in other fields of study.

We agree with the reviewer that our introduction was too neurocentric. In light of their concern, together with their major criticism above, and minor point #6 below, we felt that it made sense to streamline the introduction. The revised introduction is significantly shorter, mentions examples across life sciences (physiology, cancer research, plant biology, and neuroscience), and focuses on ease of production, and the educational nature of our work.

The previous references, together with a discussion of other applications of two-photon microscopy, have been moved to a new section in the discussion. As we are no experts in these ancillary fields, we do not want to misrepresent their work and felt it would be best to do this in the format of a discussion.

2. I suggest to provide an estimation of the budget required to purchase a Ti:S laser, as this can dramatically change the final costs.

We now mention that, currently, a Ti:Sapphire system is priced two to three times higher than a fixed wavelength fiber laser.

3. Line 82: please provide an estimation of the distortion percentage.
This was added.

4. Line 272: please provide an estimation of the cost.
This was added with a note that we refer to US-$ in 2024.

5. Line 324: please reformulate the sentence "Confident that the system operates as intended".
This half-sentence was deleted.

6. In the discussion session, please provide an overview of the possibility to use the setup to perform ex-vivo studies on explanted organs.

We appreciate the reviewer’s comment. The original discussion contained four sections:

- Improvements to the microscope,
- teaching advanced methods in neuroscience,
- open scientific instrumentation, and

- a disclaimer.

To us, adding a section focusing on "*ex-vivo studies on explanted organs*" did not seem appropriate. However, taking the reviewer's comment in good faith, together with their concerns above, we decided to remove most of the application examples of two-photon microscopy from the introduction, and add a "*scientific use cases*" section to the discussion. This new section now includes many examples for *ex vivo* experiments.

In addition, we list several improvements to the system in the "*Improvements to the microscope*" section that would make our microscope particularly useful for *ex vivo* imaging work.

Reviewer #2: In this manuscript, the Authors describe a two-photon laser scanning fluorescence microscope that is cost-effective, easy to build, and well-suited for both didactic and scientific imaging purposes. Additionally, the Authors demonstrate how this open-source microscope can be utilized as a teaching tool.
The manuscript is well-written and scientifically sound, making it a valuable contribution to the bioimaging community. I particularly appreciate how the manuscript covers technical aspects in a manner accessible to students and early-career researchers while maintaining a level of rigor that more experienced researchers will also find inspiring and thought-provoking.
I recommend the Editor accept this manuscript for publication, pending very minor revisions.

Specific suggestions for revision:

1. The in-series configuration of the two-channel power supply to provide +28 V and -28 V should be described in greater detail. For instance, the connections needed to generate these opposing voltages, along with the shared ground, should be clearly explained. While this information is briefly mentioned in the supplementary materials, a more detailed description would enhance clarity.

We have added a new paragraph to the main article and supplement, and revised Supplemental Fig. 11 to illustrate the wiring logic. We hope that this adds sufficient clarity.

2. I understand that the Authors use the assembly composed of a half-wave plate and a polarizing beam splitter to share the same light source between two different setups. However, this configuration, described also in the "didactic" section of the supplementary materials, leads to a situation where the control of the laser power is redundantly delegated to two different subsystems: this assembly and the Pockels cell. This would not be an issue in a general sense, but it conflicts with the nature of a simple and cost effective (so, almost minimalist) setup.

We thank the reviewer for this comment. It escaped our attention that this could be confusing. To be explicit here as well, the half-wave plate and beam splitter are not used to control the laser's power. Their purpose is exclusively to split the beam into two beams, each with 50% of the power. This is not adjusted further. All power control of the microscope itself is done by the subsequent EOM + shutter. We revised **Figure 1** to make this clear. We also revised the accompanying text to make explicit that while these are optional parts, in many applications a single laser can easily power two imaging systems at the same time. With cost in mind, this can be strategic.

3. Some components appear in the CAD visualization of the setup but not in the cartoon visualization of the setup (eg. the beam block and the beam splitter in Fig. 1a and 1b). This discrepancy could cause confusion for readers, and harmonizing these figures would improve clarity.

We thank the reviewer for taking the time to explore our CAD drawings :-). This concern, together with #2 above, motivated us to redesign **Figure 1**. The revised cartoon contains all elements from the CAD

design. For clarity, we split the elements into two logical groups: One for beam conditioning on the table. And one for the microscope head itself, beginning at the scanners.

4. On lines 213 and 214: non-SI units (feet) are used instead of SI units. These should be converted for consistency.

We have added the metric dimensions as well. In our part of the world, unfortunately, measurements in feet for table sizes are common practice.

5. On lines 463 and following, point (6): it would be beneficial to mention that an IR LED is also needed to provide illumination for the wide-field configuration.

This was added.
